# Stressor Factors for Spanish Nursing Students in a Pandemic Context: An Observational Pilot Survey

**DOI:** 10.3390/nursrep12040070

**Published:** 2022-09-30

**Authors:** Silvia Reverté-Villarroya, Elsa Gil-Mateu, Esther Sauras-Colón, Josep Barceló-Prats, Núria Albacar-Riobóo, Laura Ortega

**Affiliations:** 1 Department of Nursing, Universitat Rovira I Virgili, 43500 Tarragona, Spain; 2 Instituto de Investigación Sanitaria Pere Virgili, Hospital Verge de la Cinta de Tortosa, 43500 Tortosa, Spain

**Keywords:** students nursing, education, nursing, stress, psychological, pandemic, clinical skills, patient care

## Abstract

Background: The context of the pandemic in Spain meant a high demand for care. The purpose of this pilot work was to determine the stress factors, conducted on final-year nursing students at a Spanish university, who volunteered to carry out healthcare tasks, in pandemic and post-pandemic contexts. Methods: An observational prospective cohort pilot survey was conducted with an intentional sampling of the forty-seven students. We collected sociodemographic and stressor data using the validated KEZKAK questionnaire. The STROBE checklist was used to evaluate the study. Results: The median scores obtained from nursing students incorporated as auxiliary health workers are lower than those who were not incorporated, and statistically significant differences were found: lack of skills and abilities (*p* = 0.016); relationship with tutors and colleagues (*p* = 0.004); impotence and uncertainty (*p* = 0.011); inability to manage the relationship with the patient (*p* = 0.009); emotional involvement (*p* = 0.032); distress caused by the relationship with patients and item overload (*p* = 0.039); and overload items (*p* = 0.011). The post-pandemic only maintained “lack of skill and abilities” (*p* = 0.048), from nursing students incorporated as auxiliary health workers. Conclusion: This pilot study showed that nursing students who joined as auxiliary health personnel presented less perceived stress than non-incorporated nursing students. Still, more prospectively designed clinical research is needed.

## 1. Introduction

The context of the SARS-CoV-2 pandemic led to the adoption of extraordinary and unprecedented measures in all countries around the world, and Spain was not an exception [1]. The sudden high demand for assistance in hospitals throughout the country, along with the high percentage of infected healthcare professionals (19.6%), led to the Government of Spain issuing ministerial order SND/232/2020 on 15 March of 2020, authorizing the hiring of final-year nursing students as auxiliary health staff to provide healthcare support under the supervision of professional nurses [1,2].

The pandemic, which led to a mandatory lockdown and the closure of universities, forced the academic world to adopt virtual teaching. The clinical traineeships of university students in health sciences, such as nursing and medicine, were interrupted by the decision of the regional governments or the universities [3], and later on by ministerial order [4]. As commented above, the Spanish government authorized the legal incorporation of final-year students of health careers in jobs intended for health care [5], and adhering to these regulations, many students entered the healthcare workforce without completing their training, in a context of uncertainty of their final evaluation or how they would achieve the academic skills required to complete their undergraduate studies. Additionally, they were concerned about catching the virus and/or transmitting it to their families, as reported in other pandemic studies [6]. Nevertheless, some students accepted these jobs because of social commitment, vocation, and professional ethics [7]. 

Although, there are many stress-inducing factors, it seems that in the last stage of the degree, most of the stress-inducing factors are academic, such as examinations, and factors related to clinical clerkship are also involved, such as a perceived lack of the knowledge and necessary skills to care for patients or having to work under the close supervision of tutors [8,9]. It is known that clinical is one of the most stressful stages for nursing students, as they experience the reality of healthcare for the first time as professionals [10,11]. Some studies indicate that students experience high levels of stress, depression, and anxiety, especially when approaching graduation [12,13], and specific factors have been identified as highly stressors, such as confusion of medication, risk of infection through a needle stick, and making mistakes [14]. According to the facts already presented, the purpose of this pilot work was to determine the stress factors, through an observational, prospective cohort study conducted on nursing students at a Spanish university, who volunteered to carry out healthcare tasks, in pandemic and post-pandemic contexts and, if possible, further test the hypothesis that the nursing students voluntarily incorporated to work due to the pandemic present a lower perceived total stress than non-incorporated students.

## 2. Materials and Methods

An observational, prospective cohort pilot survey design was carried out with an intentional sample of a total of forty-seven students enrolled in the clinical practice subjects of the last nursing year of the Rovira Virgili University, framed within the Terres de l’Ebre health region, Catalonia, Spain [15]. There were no exclusion criteria. The total sample agreed to participate. 

The prospective study was carried out in two periods; the beginning of the state of alarm of the SARS-CoV-2 pandemic “pandemic context” in March 2020, and the end of the state of alarm in May 2021 “post-pandemic context”. The Strengthening the Reporting of Observational studies in Epidemiology (STROBE) guidelines were conducted in this observational study.

### 2.1. Instruments

We collected the following sociodemographic data, according to the literature review and expert opinion: age, gender, incorporation to work tasks with health aid contract (yes/no), as well as the area of incorporation in the health system due to the pandemic. The KEZKAK (“Worries”, in the Basque language) questionnaire [16] was used to measure stressors during clinical clerkship. This is a self-administered and validated scale adapted to Spanish nursing students. 

The questionnaire consists of 41 items and assesses the level of concern about each situation described (0: not at all; 1: little; 2: quite a lot; 3: a lot), grouped into 9 factors, and each factor includes the following items: Factor 1 = Lack of skills and abilities, Items 15, 16, 13, 4, 2, 6, 26, 1, 3, 17 and 5 (maximum score 33); Factor 2 = Contact with human suffering, Items 27, 39, 18, 10, 9, 31, 32, 29, 14 and 34 (maximum score 30); Factor 3 = Relationship with tutors and peers, Items 12, 28, 19, 25, 1 and 20 (maximum score 18); Factor 4 = Impotence and uncertainty, Items 23, 38, 14, 41, 36, 6, 32, 3, 17, 2 and 20 (maximum score 33); Factor 5 = Inability to manage the relationship with patients, Items 5, 33, 7, 30, 29, 39, 17 and 20 (maximum score 24); Factor 6 = Emotional involvement, Items 21, 8, 22 and 31 (maximum score 12); Factor 7 = Distress caused by relationship with patients, Items 11, 24, 26, 15 and 14 (maximum score 15); Factor 8 = Patient seeking an intimate relationship, Items 40 and 37 (maximum score 6); Factor 9 = Overload, Items 35, 36, 34, 30, and 31 (maximum score 15).

The high scores in the total scores show which students are concerned, and to what extent the situations in clinical clerkships can be stressful. The factorial scores point toward specific aspects of high stressors. The total scores range from 0 to 123. We obtained a global score that represents the degree of stress that students experience during the workforce tasks, as well as a score for each of the nine factors. We calculated the psychometric characteristics of the sample using Cronbach’s alpha for these items at incorporation at lockdown stage (α = 0.920) and the deconfinement (α = 0.959).

### 2.2. Procedure

The KEZKAK questionnaire was sent to all participants through the Rovira Virgili University Moodle virtual platform in all stages of the study. The students were able to complete the questionnaire through a web link or Quick Response Code, using a computer, tablet, or smartphone. All participants received the online survey and an invitation with detailed information on the purpose, anonymity, and confidentiality of this study. Consent was implied if the participants connected to the website link and completed the questionnaire.

### 2.3. Statistical Analysis

We carried out the descriptive analysis of the qualitative variables using the absolute frequency and the corresponding percentage. Quantitative variables were described using the median and interquartile range (IQR). Data normality was studied with the Shapiro–Wilk test. Qualitative variables’ differences were calculated using the Chi-square test. The Mann–Whitney U test was used to analyze the differences between the groups of students according to their decision of incorporation as healthcare personnel or not. Correlation between KEZKAK scores and demographic characteristics at the two assessment points were analyzed with Spearman correlation. All analyses were carried out with the SPSS Statistics v.27 software (IBM) and the established level of statistical significance was *p* < 0.05.

## 3. Results

There were no significant differences in age, gender, or incorporation distribution between the two groups. The median age of the study population was 22 years, ranging from 21 to 24 years, and 86.2% were women. A total of 31 (66%) students entered the workforce just as the Spanish government declared a state of alarm “pandemic context”. We obtained seven missings in the end-of-alarm state assessment “post-pandemic context”. The demographic and incorporation data of the sample are described in Table 1.

Table 2 shows data regarding KEZKAK scores in the two assessment points.

KEZKAK scores significant differences were observed between the nursing students incorporated and not incorporated as auxiliary health workers in the two periods studied. The median scores obtained from nursing students incorporated as auxiliary health workers are lower than those who were not incorporated. The statistically significant dimensions between the incorporated students versus those not incorporated at the beginning of the alarm state are factors 1 “lack of skills and abilities” (*p* = 0.016), factor 3 “relationship with tutors and peers” (*p* = 0.004), factor 4 “impotence and uncertainty” (*p* = 0.011), factor 5 “inability to manage the relationship with workmates” (*p* = 0.009), factor 6 “emotional involvement” (*p* = 0.032), factor 7 “distress caused by the relationship with patients”(*p* = 0.039), and factor 9 “overload items” (*p* = 0.011). In the post-pandemic context, forty nursing students responded, with a response loss of seven (14.9%). 

The statistically significant dimension at the end of the alarm state that was maintained was the stressor’s perception of factor 1 “lack of skill and abilities” (*p* = 0.048), also finding higher scores in the non-incorporated group. Although the median of the scores was higher in the non-incorporated group in the two periods, students in both groups expressed a high level of stress. Regarding the total questionnaire score, we did not observe significant differences between the periods (Table 2).

Correlation between two variables (age, and incorporation), and KEZKAK’s total score are presented in Table 3. We found that the KEZKAK score was significantly correlated with the incorporation of nursing students as auxiliary health workers during the pandemic (*p* = 0.003). Those who were incorporated as auxiliary health workers scored significantly lower in the pandemic context. In addition, no differences in KEZKAK’s total score were found between men and women in either a pandemic (*p* = 0.823) or post-pandemic context (*p* = 0.379) (data not shown).

## 4. Discussion

This pilot survey aimed to determine stress factors through a prospective, observational cohort study conducted in final-year nursing students. The results were compared with students who volunteered to perform healthcare tasks and those who did not in pandemic and post-pandemic contexts. The results of our study show that nursing students who experienced the pandemic in the final year of their studies reported lower scores on the KEZKAK questionnaire. They perceived a lower risk of suffering stress than their counterparts who did not have this experience. Our findings suggest that those students who performed healthcare tasks during the pandemic have developed better coping strategies, which could lead us to conclude that experience was partly positive perceived. 

The pandemic has been shown to have greatly impacted the health of a large part of the population, affecting not only physical well-being, but also psychological well-being and mental health [17,18]. Students in their senior year have been particularly exposed to this stressful situation. More than half of our student population entered the workforce without completing their training and had to keep up with academic activities while working in a contagious environment and an over-strained healthcare system, which are themselves considered stressful elements [19,20]. 

In our study, 66% of the sample volunteered to carry out healthcare relief tasks during pandemic context. The main perception of stress from nursing students hired for health care was “lack of skills and abilities”, both in the pandemic and post-pandemic context. The perception of lack of competence is a stressful factor that tends to decrease with experience. This could have been influenced by having to combine health care work with unfinished university studies [21]. One of the reasons that could explain why this dimension is still present in the post-pandemic context is the short time elapsed between the evaluations. On the other hand, a lack of skills is normal, when nurses feel unable to care for patients as they would in normal situations [20,22]. In addition, students have been able to experience various situations new to them, which have made them feel lacking in competence or ability to tackle them. We have also found that our students presented significant differences, although with low scores in the “relationship with tutors and peers” in the pandemic context, which is associated with students’ perception of poor integration in the healthcare team [23,24]. 

Students often express their concerns about feeling accepted by their tutors, since facing an unknown environment usually causes fear and concerns about their ability to offer proper care as well as other difficulties. These concerns often derive from the relationships between patients and other professionals. Positive and negative emotions are part of nursing care and the learning process. An important aspect of students’ first experiences as professional nurses is learning to manage emotionally distressing situations [25]. Added to this is the pressure of being evaluated [26,27,28]. Although the dimension “contact with human suffering” did not show significant differences, we observed high stress scores in relation to the rest of the dimensions. Our students may have experienced situations such as facing the death of their patients who, apart from having experienced an isolated hospitalization situation, have probably died alone without their relatives, the risk of infecting themselves or the fear of infecting their own relatives, and the lack of resources, such as personal protective equipment, between other stressful scenarios [17,29]. These facts could also explain the presence of the stressor “impotence and uncertainty”.

Other stress factors with high scores found in the pandemic context were “inability to manage the relationship with patients”, “emotional involvement”, and “distress caused by the relationship with patients”. These factors have already been reported in the literature as stressors that can be grouped together, as they imply a lack of self-confidence [10,30]. These types of stressors may be related to the pandemic context, a fear of making mistakes, feeling impotent, ignored, or self-conscious [31]. Nurses are in permanent contact with the suffering of patients and experience grief, pain, and compassion alongside them [25]. These experiences may have increased the stress caused by emotional involvement [32]. Recent studies about the professionals who worked during the confinement stage of the pandemic describe how the above findings may be associated with a high risk of developing symptoms of mental health issues [33,34]. All this, could have affected the stress levels reported by our students.

Work overload is usually the main source of stress for students and nursing professionals [18,35]. Perhaps, in this case, the stress due to “overload items” was heightened by the uncertainty and unfamiliarity of the situation to students who were still undergoing academic training and were expected to combine it with their clinical clerkship. It should be noted, however, that students who did not join clinical traineeships reported a higher level of stress than the students who did join, which is probably related to mandatory social distancing and the influence of the media [29]. Likewise, it should be noted that other variables related to emotional well-being, such as sense of coherence, were not assessed in this study, and recent studies have shown that good levels of sense of coherence contribute to better mental health and may even improve nurses’ ability to withstand stress [18,36]. 

Finally, the median of total scores obtained about the self-perception of the stress from nursing students incorporated as auxiliary health workers was lower than those who were not incorporated at the pandemic and post-pandemic context. Thus, our results seem to show that those students who did not experience the pandemic had a more stressful situation.

To sum up, the findings of our study provide more information on perceived stress from the interpretation of the dimensions of the validated KEZKAK questionnaire of students incorporated as health care workers, which can help us design effective coping strategies and implement stress-management techniques that focus on building resilience and minimizing the psychological impact of stress [37,38]. 

Despite all the above, our study has some limitations. This is a small cohort pilot survey, with purposive sampling, and included fourth-year nursing students from only one university, which limits its external validity and the ability to draw firm conclusions. Another limitation was that the reasons for early non-engagement in the health care system were not collected, so other possible personal variables, such as coping skills, emotional response to stress, and unknown family or environmental issues, which could intensify existing stressors, must be taken into account. It should also be noted that the impact of pandemic in the Terres de l’Ebre (Catalonia, Spain) non-urban health region was less than in other regions in the country, perhaps due to its geographical dispersion, as it reduced transmission by contact [39,40]. Lastly, we collected the data through a self-administered survey. Although this method is greatly accepted by the scientific literature, it is subjective, and factors such as vulnerability when completing the survey may result in biases [41]. However, as it is reported in the first person, the self-administered questionnaire provides detailed information about personal experiences. That said, self-reported measures tend to provide very accurate information and offer one of the most realistic approximations of what the person who is being surveyed experiences firsthand. 

## 5. Conclusions

The present study reveals the factors that caused the most stress to nursing students in the pandemic and post-pandemic context. Our study shows that those who volunteered to work after the pandemic showed less perceived stress than those who did not. Therefore, we believe that early on boarding may have brought them closer to reality and improved their self-perceived stress. It is also noteworthy that the group of students who experienced the sudden incursion of the pandemic entered the healthcare system in better circumstances after experiencing the pandemic outbreaks. 

Although the results are exploratory, despite the discomfort the pandemic caused these students, their early entry may have provided better coping strategies. Consequently, beginning in undergraduate training, students should be taught to integrate effective coping strategies designed to provide the necessary resources for managing potential stressors, building resilience, and minimizing the psychological impacts of stress. Still, further clinical, prospectively designed research is needed in other similar cohorts, and settings are needed. 

## Figures and Tables

**Table 1:** Demographic characteristics and incorporation or not into health system due to the context of the pandemic in Spain.

Demographic Characteristics	Pandemic Context (*n* = 47)	Post Pandemic Context (*n* = 40)	*p*-Value
**Age Median [IQR]**	23 [21–25]	22 [21.25–24]	0.687
**Gender** **Male** **Female**	7 (14.9%) 40 (85.1%)	5 (12.5%) 35 (87.5%)	0.747
**Incorporation** **Yes** **No**	31 (66.0%) 16 (34.0%)	29 (72.5%) 11 (27.5%)	0.511

Age is expressed using the median (IQR). Qualitative variables (gender and incorporation) are expressed using absolute frequency (percentage).

**Table 2:** Differences between the stressors expressed by the students who were incorporated or not into the workforce during the pandemic context and post-pandemic context.

	Pandemic Context (*n* = 47)			Post Pandemic Context (*n* = 40)			* *p*-Value
	Incorporation (*n* = 31)	No Incorporation (*n* = 16)	*p*-Value	Incorporation (*n* = 29)	No Incorporation (*n* = 11)	*p*-Value	
Factor 1. Lack of skills and abilities	23 [16–26]	26 [23.25–29.75]	0.016	25 [20–26.5]	27 [23–29]	0.048	0.359
Factor 2. Contact with human suffering	22 [16–24]	25 [20.75–26]	0.065	21 [13.5–25.5]	22 [15–26]	0.378	0.184
Factor 3. Relationship with tutors and peers	10 [8–14]	14.5 [12.5–16.75]	0.004	12 [8.5–15]	16 [10–18]	0.196	0.361
Factor 4. Impotence and uncertainty	25 [21–28]	27.5 [26–31]	0.011	26 [23–29.5]	28 [23–31]	0.504	0.627
Factor 5. Inability to manage the relationship with workmates	17 [14–20]	20 [17.5–21]	0.009	17 [14–20]	18 [16–22]	0.247	0.090
Factor 6. Emotional involvement	8 [6–10]	10 [8.25–11]	0.032	8 [6–10.5]	10 [7–11]	0.184	0.199
Factor 7. Distress caused by relationship with patients	9 [6–13]	11.5 [9.25–13]	0.039	9 [6.5–11]	10 [7–13]	0.368	0.313
Factor 8. Patient seeking an intimate relationship	4 [2–5]	5 [4–5.75]	0.087	4 [1.5–4.5]	4 [2–6]	0.435	0.342
Factor 9. Overload Items	10 [9–12]	12 [11–13.75]	0.011	11 [7.5–13]	11 [9–13]	0.582	0.443
Total Score	74 [52–90]	83.5 [70.25–93]	0.106	66 [61–90]	71 [60–83]	0.596	0.206

The score for each factor of the KEZKAK instrument is expressed with the median and (IQR). * *p*-value (differences in the scores of the two different assessment points: State of alarm “Pandemic context”/End of state of alarm “Post pandemic context”).

**Table 3:** Correlation between demographic characteristics and KEZKAK total score in a pandemic and post-pandemic context.

	KEZKAK Score Pandemic Context (*n* = 47)	KEZKAK Score Post-Pandemic (*n* = 40)
Age Spearman’s rank correlation coefficient *p*-value	−0.102 0.496	0.091 0.597
Incorporation as auxiliary health worker Spearman’s rank correlation coefficient *p*-value	−0.429 0.003 *	−0.187 0.249

* *p* < 0.05.

## Data Availability

The data are available upon reasonable request.
